# Recent advances in NAFLD: current areas of contention

**DOI:** 10.12703/r/12-10

**Published:** 2023-05-02

**Authors:** Erica Jennison, Christopher D Byrne

**Affiliations:** 1 Chemical Pathology, University Hospital Southampton NHS Foundation Trust, Southampton, UK; 2 Human Development and Health, Faculty of Medicine, University of Southampton, Southampton, UK; 3 National Institute for Health and Care Research, Southampton Biomedical Research Centre, University of Southampton and University Hospital Southampton National Health Service (NHS) Foundation Trust, Southampton, UK

**Keywords:** NAFLD, MAFLD, liver fibrosis, metabolic syndrome, type 2 diabetes

## Abstract

This brief review focuses on two contentious issues within the field of non-alcoholic fatty liver disease (NAFLD); the first is the recent effort to redefine NAFLD as metabolic (dysfunction)-associated fatty liver disease (MAFLD). The modification of “NAFLD” to “MAFLD” is expected to highlight the role of metabolic factors in the disease aetiology, which is hoped to improve patient understanding of the disease, facilitate patient-physician communication and highlight the importance of public health interventions in prevention and management. The diagnostic criteria for MAFLD allow it to coexist with other forms of liver disease, which recognises that metabolic dysfunction contributes towards disease progression in other liver pathologies, such as alcoholic liver disease. However, there remain concerns that renaming NAFLD may be premature without fully considering the broader implications, from diagnostic criteria to trial endpoints; therefore, the new definition has not yet been accepted by major societies. Another contentious issue within the field is the gap in our understanding of how patients undergoing therapeutic interventions should be monitored to assess amelioration/attenuation or the worsening of their liver disease. Biomarker scoring systems (such as the ELF test and FIB-4 test) and imaging techniques (such as transient elastography [TE] and magnetic resonance imaging [MRI] techniques) are proven to be reasonably accurate, and comparable with histology, in the diagnosis of NAFLD and evaluation of disease severity; however, their use in monitoring the response of disease to therapeutic interventions is not well established. Whilst biomarker scoring systems and TE are limited by poor diagnostic accuracy in detecting moderate fibrosis (e.g. F2 liver fibrosis defined by histology), more accurate MRI techniques are not practical for routine patient follow-up due to their expense and limited availability. More work is required to determine the most appropriate method by which therapeutic interventions for NAFLD should be monitored in clinical practice.

## Abbreviations

ALT; alanine aminotransferase, CKD; chronic kidney disease, CVD; cardiovascular disease, ELF; enhanced liver fibrosis, ELPA; European Liver Patient's Association, GCKR; glucokinase regulatory protein, GLP-1; glucagon-like peptide-1, GIP; glucose-dependent insulinotropic peptide, HSD17B13; hydroxysteroid 17-beta dehydrogenase 13, NAFLD; non-alcoholic fatty liver disease, NAS; non-alcoholic fatty liver disease activity score, NASH; non-alcoholic steatohepatitis, NM-NAFLD; non-metabolic-non-alcoholic fatty liver disease, MBOAT7; membrane-bound O-acyltransferase domain containing 7, MAFLD; metabolic (dysfunction)-associated fatty liver disease, MRE: magnetic resonance elastography, MRI; magnetic resonance imaging, MRI-PDFF; MRI-proton density fat fraction, PNPLA3; patatin-like phospholipase domain-containing protein 3, SGLT2; sodium-glucose cotransporter-2, TE; transient elastography, TM6SF2; transmembrane 6 superfamily member 2, T2DM; type 2 diabetes mellitus.

## Introduction

Non-alcoholic fatty liver disease (NAFLD) is the most common liver disease, affecting around one-quarter of the population worldwide^1^. Despite the significant rising epidemic of NAFLD, limited pharmacological interventions are available for its treatment, and unlike other highly prevalent conditions, NAFLD has received little attention from the global public health community. That said, significant progress has been made in the development of biomarkers and imaging techniques to diagnose NAFLD and grade the severity of liver fibrosis. In this brief review, we have chosen to focus on two contentious issues in the field of NAFLD. The first contentious issue is the recent effort to redefine NAFLD, the impact that this may have on the population with the disease, its recognition amongst affected people and healthcare professionals, the potential to find new treatments, and interpretation of the effects of existing drugs and their efficacy in treating this fatty liver disease. The second focus of this review will highlight the current gap in our understanding of how patients undergoing therapeutic interventions should be monitored to assess amelioration/attenuation or the worsening of their liver disease. This is particularly relevant as several drugs are now used routinely in clinical practice to treat patients with type 2 diabetes mellitus (T2DM) that also have efficacy in treating liver disease in NAFLD. For these persons living with both T2DM and NAFLD, clear guidance is needed on the most appropriate method for monitoring liver disease responses to treatment.

### 1. A change in terminology from “NAFLD” to “MAFLD”

Previously, there has been criticism that the characterisation of NAFLD has led physicians to overemphasise alcohol use, and therefore underemphasise the importance of metabolic risk factors^2–4^. As a result, there is also debate over what should be considered the threshold for “significant” alcohol consumption when diagnosing NAFLD^5–7^. There has been increased recognition of NAFLD as a heterogenous disorder, with different metabolic and genetic factors involved in its pathogenesis and contributing to its progression and prognosis. It may be the heterogeneity and the imprecise definition of NAFLD that are in part responsible for the muted efficacy of many of the drugs in development for the treatment of this condition. In 2020, a group of international experts from the European Liver Patients’ Association (ELPA) reached a consensus that NAFLD does not reflect our current understanding of the disease and a more accurate term would be metabolic (dysfunction)-associated fatty liver disease (MAFLD)^8^. The modification of “NAFLD” to “MAFLD” is expected to highlight the role of metabolic factors in the disease aetiology, which is hoped to improve patient understanding of the disease, facilitate patient-physician communication and highlight the importance of public health interventions in prevention and management.

**Figure 1.**
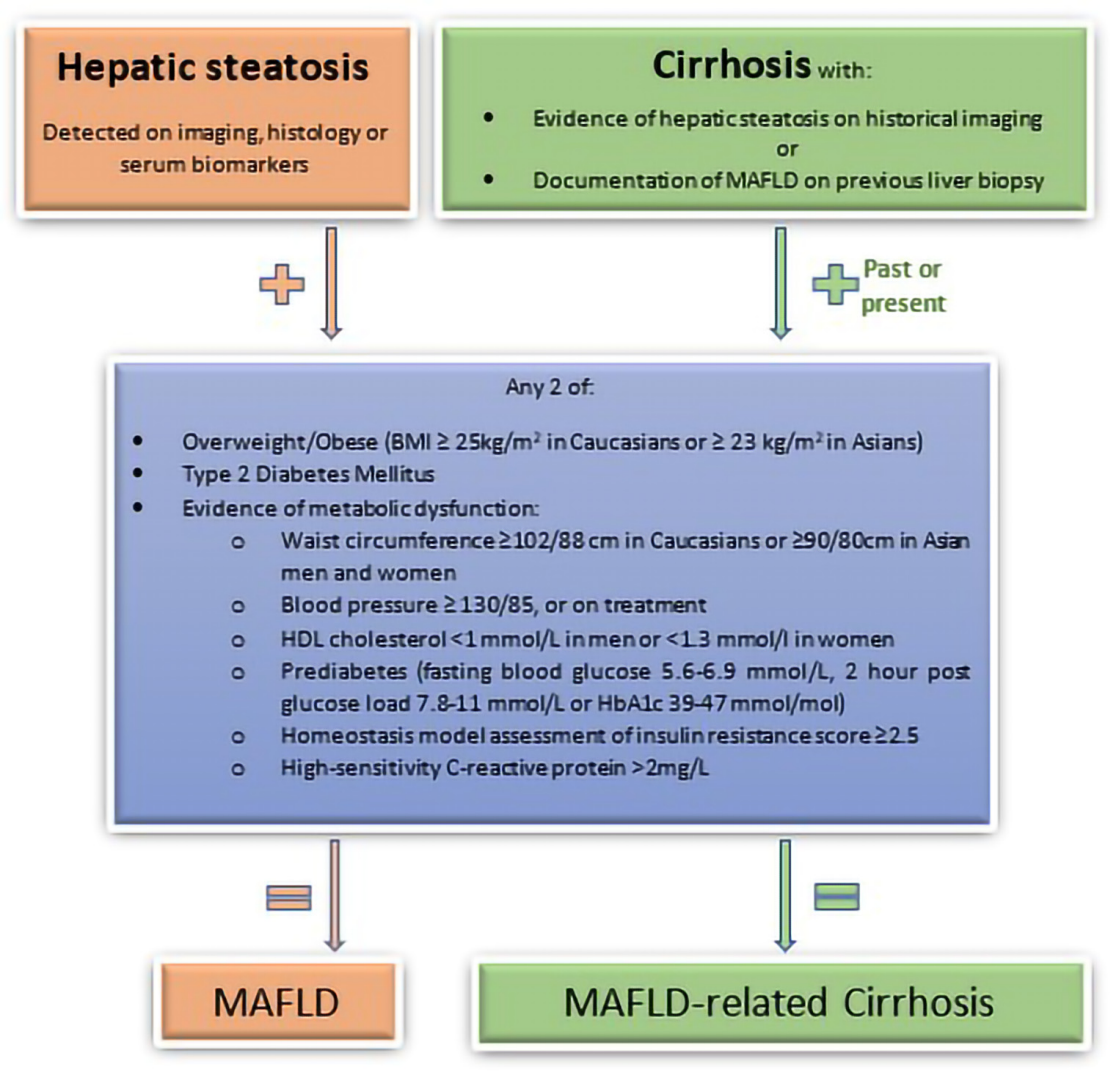
Flow chart for the proposed diagnostic criteria for MAFLD and MAFLD-related cirrhosis. Adapted from 8.

Unlike NAFLD, the diagnosis of MAFLD does not require the absence of other secondary causes of hepatic steatosis and is based only on positive diagnostic criteria (see Figure 1). The diagnosis of MAFLD is made by the presence of hepatic steatosis (detected by serum biomarker scores, imaging techniques or histology) and at least one of the following: (a) overweight/obesity; (b) T2DM; or (c) metabolic dysregulation (requiring at least two of the metabolic abnormalities described in Figure 1)^8^. Diagnostic criteria for MAFLD emphasise the importance of metabolic dysfunction in contributing to hepatic steatosis, regardless of other potential aetiologies, and allows MAFLD to coexist with other liver diseases. Furthermore, MAFLD criteria may identify the presence of metabolic dysfunction in those with “lean-NAFLD”, a disorder that previously may have caused diagnostic dilemmas. The ELPA also proposed that if patients with cirrhosis meet the specified criteria outlined in Figure 1, then they should be diagnosed with MAFLD-related cirrhosis, and the term “cryptogenic cirrhosis” in these individuals should be avoided^8^.

MAFLD coexisting with other liver diseases is defined as dual aetiology fatty liver disease and is likely to be highly prevalent amongst all causes of liver disease, given the rising epidemic of metabolic dysfunction. For example, the prevalence of obesity and metabolic syndrome in alcoholic liver disease (ALD) is as high as 44.5% and 32.4%, respectively^9^. Allowing MAFLD to coexist with other forms of liver disease recognises that these pathologies often work synergistically to progress liver dysfunction. For example, a number of population-based prospective studies and patient cohort studies have provided evidence that obesity, T2DM and metabolic syndrome can exacerbate the progression of ALD and also increase hepatocellular carcinoma incidence and mortality^8–10^.

A further statement from the ELPA suggests that disease severity should be described by the grade of activity and the stage of fibrosis, in a manner similar to that done for other chronic liver diseases^8^. The dichotomous stratification of NAFLD into steatohepatitis and non-steatohepatitis may not capture the full spectrum of the disease course, particularly in response to pharmacological interventions. This shift in severity grading is hoped to help case identification and improve the way therapeutic interventions are monitored. However, as ongoing clinical trials were designed to account for the current severity stratifications, abandoning the term “steatohepatitis” could lead to potential derailment of active research.

***The impact of a change in both classification and diagnostic criteria from “NAFLD” to “MAFLD”*.** A shift in diagnosis from “NAFLD” to “MAFLD” will change the population with the disease. In two large cross-sectional studies of the general population, the prevalence of NAFLD and MAFLD were 37.1% and 39.1%, respectively, in the North American population, and 28% and 37.3%, respectfully, in the South Korean population^11,12^. The majority of subjects with MAFLD that did not meet the NAFLD criteria in these studies consumed significant alcohol or had viral hepatitis. A small proportion of patients with NAFLD will not meet the criteria for MAFLD and can be termed non-metabolic-NAFLD (NM-NAFLD). The proportion of NM-NAFLD varies among studies and may represent true population differences; in three separate studies, the proportion of individuals with NAFLD who were classified as NM-NAFLD were <1% in North America^13^, 6.3% in China^14^, and 15.3% in Japan^15^.

The difference between the severity of liver disease in NAFLD, MAFLD and NM-NAFLD is not clear. Huang *et al.* found a similar severity of liver fibrotic burden (based on biochemical markers) between 4,087 patients with MAFLD and 46 with NM-NAFLD^13^. Conversely, a Taiwanese study, using histological findings, showed that 42.8% of patients with MAFLD presented with advanced liver fibrosis, while none of the subjects with NM-NAFLD did^16^. Yamaura *et al.* found that patients with MAFLD, but without NAFLD, had significantly higher fatty liver index scores, NAFLD fibrosis scores, and liver stiffness assessed by transient elastography (TE)^15^.

NAFLD is a multisystemic disease associated with an increased risk of T2DM, cardiovascular disease (CVD) and chronic kidney disease (CKD)^17^. Owing to the metabolic dysfunction required for the diagnosis of MAFLD, and the potential presence of coexistent pathologies, such as viral hepatitis and alcohol excess, it could be expected that the MAFLD population, compared to the NAFLD population, would be burdened by more extrahepatic disease. Very few studies have explored the different prevalence of extrahepatic disease between the NAFLD, MAFLD and NM-MAFLD populations, and current data are not conclusive. CVD is the most common cause of death in individuals with NAFLD, and several studies have shown the risk to be even higher in patients diagnosed with MAFLD^12,18,19^. Huang et al. found that MAFLD increased the risk for all-cause mortality by a greater magnitude than NAFLD; however, this association was not confirmed after adjusting for metabolic parameters^20^. A recent Chinese study found that MAFLD was associated with worse renal outcomes compared to NM-NAFLD^14^.

NM-NAFLD represents a distinct disease with a pathogenesis, prognosis and therapeutic strategy that are likely to be different to that of MAFLD. The identification of individuals with NM-NAFLD will allow further research into the aetiology and most appropriate management of this disease, an area that is still very poorly understood. Several secondary factors have been implicated in the aetiology of NAFLD that may be particularly relevant in the pathogenesis of NM-NAFLD, such as high fructose intake, protein malnutrition, the gut microbiome, steatogenic drugs and genetic predisposition^21–23^. The genes involved in the development of NAFLD, are related to the regulation of lipid metabolism in the liver and include patatin-like phospholipase domain-containing protein 3 (PNPLA3), transmembrane 6 superfamily member 2 (TM6SF2), glucokinase regulatory protein (GCKR), membrane bound O-acyltransferase domain containing 7 (MBOAT7) and hydroxysteroid 17-beta dehydrogenase 13 (HSD17B13)^24^. Gene polymorphisms, changes in messenger RNA expression and variable splicing of these genes influence liver disease severity and the risk of progression towards cirrhosis^25^. A number of genetic variants associated with NAFLD development have also been linked to a decreased risk of other metabolic disorders^26^ and as such may have some role in NM-NAFLD pathogenesis. For example, the NAFLD susceptibility variant GCRK P446L is known to improve hepatic glucose metabolism and induce *de novo* lipogenesis, leading to elevated triglycerides in the liver but a decreased blood glucose level, therefore having a protective role in T2DM development^24^. Conversely, some NAFLD-predisposing genetic variants are associated with an increased risk of metabolic dysfunction; for example, the NAFLD susceptibility variant TM6SF2 is associated with an increased risk of T2DM^27^.

***The benefits of a change from “NAFLD” to “MAFLD”*.** A key driver for a change in terminology and definition of “NAFLD” to “MAFLD” is to reduce the perceived trivialisation of the disease. Several studies have reported that the majority of patients with NAFLD are unaware that they have the disease, and that those who are aware tend to trivialise their condition, with most indicating they are not concerned by the diagnosis^28–30^. Patients have expressed unhappiness with the term NAFLD, mostly because it contains the word "alcohol."^31^ According to a recent survey of 191 professionals, 96% of physicians had a substantial lack of knowledge regarding the differences between NAFLD and non-alcoholic steatohepatitis (NASH)^32^. Renaming the condition to “MAFLD” is hoped to increase awareness of the disease and emphasise the metabolic contribution, leading patients to recognise the importance of lifestyle changes in treating the condition. The new definition of MAFLD is expected to engage and establish better collaboration with other healthcare groups involved in the care of patients with metabolic disease (such as T2DM, CVD and CKD)^33^. By focusing on the metabolic aspects, the acronym highlights that the disease is potentially preventable and emphasises the need for appropriate resource allocation and effective public health policy decisions, an area which, despite the significant human, social and economic burden of the disease, is felt to be lacking. The pharmaceutical industry may be affected by changing the terminology and definition of NAFLD to MAFLD, which could encourage shared funding with other metabolic illnesses and result in the implementation of efficient system-wide therapies.

***The barriers to a change from “NAFLD” to “MAFLD”*.** There are concerns that renaming NAFLD may be premature without fully considering the broader implications, from diagnostic criteria to trial endpoints. According to several experts, the MAFLD definition does not adequately account for genetic steatosis, does not resolve many of the ambiguities present in the NAFLD classification, and does not improve patient risk stratification^34^. There is concern that as our understanding of the natural history, aetiology, and management of NAFLD continues to improve, we may find that the term “MAFLD” is not representative of the disease, and therefore some experts feel we should wait until we have a greater understanding of the disease before making any changes to the definition and terminology. Since this change has not been fully accepted by major societies, future studies are required to confirm the feasibility of this novel terminology. We also consider that it is really important to reach a consensus on the MAFLD criteria and how reclassifying this fatty liver disease affects diagnosis, treatments, and extrahepatic complications of this fatty liver disease.

### 2. Methods of monitoring the response of NAFLD to therapeutic intervention

Although liver biopsy is considered the gold standard for assessing disease activity and severity in NAFLD, its invasiveness, expense, unpredictability in sampling and interpretation, and all of these drawbacks prevent its widespread use in clinical practice to screen for advanced fibrosis, track the course of the illness, and assess therapeutic response in patients with NAFLD^35^. Intense research has been done to find non-invasive, repeatable, and reliable methods to fulfil these unmet clinical demands and economically viable biomarkers and imaging techniques for diagnosing NAFLD and assessing disease severity. The majority of these biomarkers and imaging techniques are proven to be accurate, and comparable with histology, in the diagnosis of NAFLD and evaluation of disease severity; however, their use in monitoring the response of disease to therapeutic intervention is less well established. This is particularly relevant as several drugs, such as pioglitazone and glucagon-like peptide-1 (GLP-1) receptor agonists are already being used in individuals with NASH and coexistent T2DM without clear guidance on the most appropriate method for monitoring liver disease in these patients^36^. pioglitazone and GLP-1 receptor agonists have been shown to improve histological features of NASH (i.e., steatosis, ballooning, lobular inflammation) or achieve resolution of NASH without further deterioration of fibrosis and therefore are widely used in patients with NAFLD and coexistent T2DM^36^. New agents such as tirzepatide, a dual GLP-1 and glucose-dependent insulinotropic peptide (GIP) receptor agonist, are showing potential promise for their ability to improve liver disease and, in our opinion, are likely to play a future role in the management of NAFLD. In a sub-study of the randomised phase 3 SURPASS-3 trial, tirzepatide significantly improved liver steatosis (assessed using MRI techniques) compared to insulin in individuals with NAFLD and coexistent T2DM^37^. Sodium-glucose cotransporter-2 (SGLT2) inhibitors have been shown to reduce liver fat content (assessed using MRI techniques) but without convincing evidence of histological improvement in NAFLD and, therefore, may be a less favourable option for influencing liver disease in patients with NAFLD who have coexistent T2DM^36^. Table 1 summarises a selection of the available biomarker scoring systems and imaging techniques that have been proposed as methods for monitoring the response to therapeutic interventions in patients with NAFLD.

**Table 1.** Biochemical and imaging methods available for monitoring liver disease responses to therapeutic interventions in NAFLD. ALT; alanine aminotransferase, ELF; enhanced liver fibrosis, MRI; magnetic resonance imaging, MRI-PDFF; MRI-proton density fat fraction, MRE; magnetic resonance elastography, TE; transient elastography.

Investigation	Evidence for its use in monitoring disease response to therapeutic intervention in NAFLD	Advantages	Limitations
**Biopsy**	Used in clinical trials as the gold standard to assess disease response to therapeutic intervention in NAFLD.	• Gold standard for diagnosis of hepatic steatosis, steatohepatitis, fibrosis and cirrhosis	• False negatives • Expensive • Invasive • Not suitable for routine long-term monitoring in clinical practice
**FIB-4 Score**	Both the FIB-4 score and ALT alone have been shown to correlate with histological changes in NAFLD in response to treatment^40,41^.	• Widely available • Accurate at predicting advanced fibrosis^42^	• Intermediate score range has poor diagnostic performance • Inaccurate in those <35 years of age^43^
**ELF test**	Correlates well with histological improvement in liver disease in some trials, including the LEAN trial (liraglutide)^44^. Performed poorly in assessing disease response to pioglitazone in the PIVENS trial^45^.	• Widely available • Accurate at predicting advanced fibrosis^46^	• Intermediate score range has poor diagnostic performance^47^
**TE (FibroScan)**	Correlates well with histological changes in liver disease in response to treatment in other diseases such as antiviral treatment in Hepatitis B^48^.	• Widely available • Validated thresholds for assessing different stages of fibrosis^49^ • Controlled attenuation parameters offer a measure of steatosis • Accurate at predicting advanced fibrosis^50^	• Potential inaccuracies due to obesity, significant hepatic steatosis, hepatic congestion, biliary obstruction, liver lesions^51–55^
**MRI-PDFF**	The majority of studies have found that MRI-PDFF correlates well with histological changes in steatosis in NAFLD in response to treatment, however some have been conflicting^40,56–58^.	• Superior diagnostic accuracy compared to ultrasound techniques for assessing liver steatosis^59^. • Less affected by obesity^60^ • Non-invasive	• Not widely available • Time consuming • Expensive • Reduced accuracy if fibrosis present or severe hepatic steatosis
**MRE**	MRE correlates well with histological changes in NAFLD in response to treatment^40^.	• Superior diagnostic accuracy compared to ultrasound techniques^59^ • Less affected by obesity^61^ • Non-invasive	• Not widely available • Time consuming • Expensive • Affected by iron overload and acute inflammation^61^

***The use of biomarkers for monitoring liver disease responses to therapeutic intervention*.** The Enhanced Liver Fibrosis (ELF) algorithm is a specialist biochemical test that has superior accuracy over other simple biochemical scoring systems^38^. The algorithm combines hyaluronic acid, the N-terminal propeptide of collagen type III, and tissue inhibitor of metalloproteinase-1, three non-liver-specific serum markers of extracellular matrix remodelling and fibrogenesis and is being routinely used by both primary care and secondary care in some centres in the UK^39^. The ELF test has good diagnostic accuracy for detecting advanced fibrosis (defined by F3 & F4 fibrosis on liver histology) and is recommended by several governing bodies as the investigation of choice for ruling out advanced fibrosis^38,46,62,63^. However, the utility of the ELF test to monitor treatment response in NAFLD needs further validation, and what constitutes a clinically meaningful response remains controversial. A limitation of the ELF test, which may restrict its use in monitoring the response of NAFLD to treatment, relates to its performance in detecting less severe liver fibrosis. Although the ELF test has been validated above a certain threshold to have high diagnostic accuracy at ruling out advanced fibrosis^46^, its performance at lower thresholds to detect moderate fibrosis (e.g. F2 liver fibrosis) is hindered by poor specificity^47^.

Several trials have shown promise that changes in the ELF test score may reflect histological improvement in liver fibrosis in patients with NAFLD undergoing therapeutic intervention. The LEAN trial demonstrated that liraglutide, when compared to placebo, improved liver fibrosis on histology, which was associated with a reduction in ELF scores^44^. Two separate trials, the first involving treatment with simtuzumab and selonsertib and the second with a fibroblast growth factor-19 analogue, demonstrated that patients undergoing treatment had a reduction in their ELF score that was significantly associated with fibrosis regression on histology^64,65^. However, a post hoc analysis of the PIVENS trial demonstrated that ELF scores did not relate to improvement in fibrosis or NASH resolution in patients treated with pioglitazone, although the scores did correlate with histological improvements in those treated with Vitamin E^45^.

The FIB-4 index, consisting of four parameters (age, aspartate aminotransferase (AST), alanine aminotransferase (ALT), and platelets), is simple, cheap and available in any clinical laboratory. The diagnostic accuracy in detecting advanced fibrosis in NAFLD is superior to other simple biomarker scoring systems, such as the NAFLD fibrosis score^42^. Although data are limited regarding the utility of the FIB-4 score in assessing the response of NAFLD to therapeutic intervention, several studies have been encouraging. Changes in ALT have been shown in two randomised controlled trials to correlate well with histological changes in patients with NAFLD undergoing therapeutic intervention^41^. In a phase 2 trial investigating the efficacy of selonsertib in NAFLD, the FIB-4 score correlated with histological improvement in hepatic steatosis, fibrosis and the NAFLD activity score (NAS)^40^. A small study involving only seven patients found that treatment with an SGLT2 inhibitor improved the FIB-4 score along with histological improvements in the NAS^66^. Some studies have shown a poor association between changes in FIB-4 scores and NAFLD progression. A retrospective study of 135 patients with NAFLD found that biomarkers for NAFLD, including the FIB-4 test and the NAFLD fibrosis score, were only weakly associated with disease progression (as assessed by liver biopsy or TE) and concluded that repeated measurements of these markers should not be used to monitor treatment response in NAFLD^67^. Furthermore, a recent study in patients with T2DM showed that 22.4% of patients with advanced liver fibrosis diagnosed by magnetic resonance elastography (MRE) or TE had a low FIB-4 score <1.3^68^. These data indicate that a significant percentage of patients at risk are incorrectly classified by the FIB-4 score. A limitation of the FIB-4 score is its poor diagnostic accuracy in patients below the age of 35 years old, a particular issue within the NAFLD population, as the prevalence among the young is rapidly growing^43^.

***The use of transient elastography to monitor liver disease responses to therapeutic intervention*.** TE is widely used for the diagnosis of advanced fibrosis and cirrhosis in NAFLD^69^. TE has the advantage of being widely available, quick, non-invasive, easy to learn and well-tolerated by patients. A meta-analysis consisting of 1,047 NAFLD patients suggested that TE is excellent in diagnosing F4 fibrosis (92% sensitivity, 92% specificity) and has moderate accuracy for F2–4 fibrosis (79% sensitivity, 75% specificity)^50^. One of the biggest challenges of TE examination is the lower success rate in obese patients, which limits its utility in monitoring the response of treatment in the NAFLD population, which has a high prevalence of obesity^51^. However, the manufacturers of TE have developed an XL probe, which measures liver stiffness at a greater depth than the standard M probe (35–75 vs. 25–65 mm) and is more successful in determining liver stiffness in obese patients with NAFLD^70^. Other confounding factors, which can lead to falsely high liver stiffness using TE, include the presence of significant hepatic steatosis^52^, hepatic congestion^53^, biliary obstruction^54^, and benign or malignant liver lesions^55^.

There are few data regarding the ability of TE to monitor the response of NAFLD to therapeutic intervention. Zeng et al. demonstrated that TE was accurate, and comparable to histology, in monitoring the response of liver fibrosis to antiviral treatment in patients with hepatitis B^48^. Similar studies, but involving the NAFLD population, need to be undertaken to determine the role of TE in monitoring patients on treatment and to consider the use of TE combined with simple biomarkers in monitoring therapeutic interventions.

***The use of MRI techniques to monitor disease response to therapeutic intervention*.** Magnetic resonance imaging (MRI) techniques, such as MRI-proton density fat fraction (MRI-PDFF) and MRE, have shown superiority over ultrasound-based techniques in the evaluation of steatosis and fibrosis in NAFLD^59,71^. MRE has low failure rates, is less affected by obesity compared to TE, and has an exceptional interobserver agreement^61,72^. However, overestimation of liver stiffness and exam failure may be linked to acute inflammation and iron overload, respectively^72^. A portion of patients who might be too obese for the MRI scanner, have metallic implants that are incompatible, or are claustrophobic might not be able to endure MRI procedures.

Data from paired liver biopsy and MRI results generally support the use of MRI to monitor disease response to treatment in NAFLD, although some findings are conflicting. A single-centre study found that a 29% reduction in liver fat, as seen on MRI-PDFF, was associated with a 2-point decrease in the histological NAS score^56^. In a recent analysis of patients enrolled in a phase II study of selonsertib, MRI-PDFF and MRE changes were associated with histological improvements in hepatic steatosis and fibrosis, respectively^40^. A recent, randomised controlled trial of MGL-3196 (a thyroid hormone receptor B agonist) versus placebo found that in patients who received MGL-3196, changes in fat reduction on MRI-PDFF predicted histological resolution of NASH^57^. In contrast, a recent analysis of pooled data found that MRI changes correlated with changes in steatosis but not with changes in the resolution of NASH, inflammation, ballooning, or fibrosis^58^. A recent longitudinal prospective study showed that combining MRI-PDFF with ALT response was more effective at predicting histological improvement in NAFLD than MRI-PDFF or ALT alone, which suggests that combining different monitoring modalities could be the most effective method to monitor liver disease response to treatment in NAFLD^73^. Despite the largely encouraging performance of MRI techniques to monitor disease response to therapeutic intervention in NAFLD, its use is limited by its restriction to specialist centres, which makes MRI largely unsuitable for widespread use.

There is currently no consensus as to how patients undergoing treatment for NAFLD should be monitored, both in terms of the modality and the frequency. Furthermore, there is no consensus on what constitutes a clinically meaningful improvement in liver disease (although most would agree that a decrease in level of fibrosis would be beneficial) and what would be an indication for continuing or discontinuing treatment for NAFLD. Larger studies are required to clarify the performance of biomarkers and imaging techniques, and their correlation with histologic changes, in response to therapeutic intervention in NAFLD. Additionally, with a move from the definition of NAFLD to MAFLD, it is important that future studies not only assess changes in liver steatosis and fibrosis in response to therapeutic intervention but also analyse changes in cardio-metabolic parameters used within the definition of MAFLD, such as improvements in glycaemia (assessed with HbA1c), obesity (assessed by BMI), and other signs of metabolic dysfunction (blood pressure, waist circumference, fasting lipid profile and high-sensitivity CRP).

## Conclusion

Huge progress is being made within the field of NAFLD, from our understanding of the disease pathogenesis to the development of potential new therapeutic interventions; however, several contentious issues remain that require further clarification to optimise the best clinical care for patients with NAFLD. Although a change in terminology and definition of “NAFLD” to “MAFLD” has significant potential to improve patient care, it is first essential that a consensus on the MAFLD criteria is reached and that the implications of this change in terms of its effects on diagnosis, treatments, and extrahepatic complications are fully understood. As more therapeutic interventions for patients with NAFLD become available, it is vital that we establish guidelines on the most appropriate non-invasive method to monitor these patients in terms of modality, frequency and what constitutes a clinically meaningful improvement in liver disease.
